# Ethnic differences in cardiac structure and function assessed by MRI in healthy South Asian and White European people: A UK Biobank Study

**DOI:** 10.1016/j.jocmr.2023.100001

**Published:** 2024-01-11

**Authors:** Kelly S. Parke, Emer M. Brady, Aseel Alfuhied, Rishabh S. Motiwale, Cameron S. Razieh, Anvesha Singh, Jayanth R. Arnold, Matthew P.M. Graham-Brown, Joanna M. Bilak, Sarah L. Ayton, Abhishek Dattani, Jian L. Yeo, Gerry P. McCann, Gaurav S. Gulsin

**Affiliations:** a Department of Cardiovascular Sciences, University of Leicester and the NIHR Leicester Biomedical Research Centre, Glenfield Hospital, Leicester, UK; b Diabetes Research Centre, Leicester General Hospital, University of Leicester, Leicester, UK; c Leicester Real World Evidence Unit, Diabetes Research Centre, Leicester General Hospital, University of Leicester, Leicester, UK

**Keywords:** Cardiac MRI, Ethnic differences, South Asian, White European, Feature tracking, Cardiac volumetry, Strain

## Abstract

**Background:**

Echocardiographic studies indicate South Asian people have smaller ventricular volumes, lower mass and more concentric remodelling than White European people, but there are no data using cardiac MRI (CMR). We aimed to compare CMR quantified cardiac structure and function in White European and South Asian people.

**Methods:**

Healthy White European and South Asian participants in the UK Biobank Imaging CMR sub-study were identified by excluding those with a history of cardiovascular disease, hypertension, obesity or diabetes. Ethnic groups were matched by age and sex. Cardiac volumes, mass and feature tracking strain were compared.

**Results:**

121 matched pairs (77 male/44 female, mean age 58 ± 8 years) of South Asian and White European participants were included. South Asian males and females had smaller absolute but not indexed left ventricular (LV) volumes, and smaller absolute and indexed right ventricular volumes, with lower absolute and indexed LV mass and lower LV mass:volume than White European participants. Although there were no differences in ventricular or atrial ejection fractions, LV global longitudinal strain was higher in South Asian females than White European females but not males, and global circumferential strain was higher in both male and South Asian females than White European females. Peak early diastolic strain rates were higher in South Asian versus White European males, but not different between South Asian and White European females.

**Conclusions:**

Contrary to echocardiographic studies, South Asian participants in the UK Biobank study had less concentric remodelling and higher global circumferential strain than White European subjects. These findings emphasise the importance of sex- and ethnic- specific normal ranges for cardiac volumes and function.

## Graphical Abstract

**Figure:**
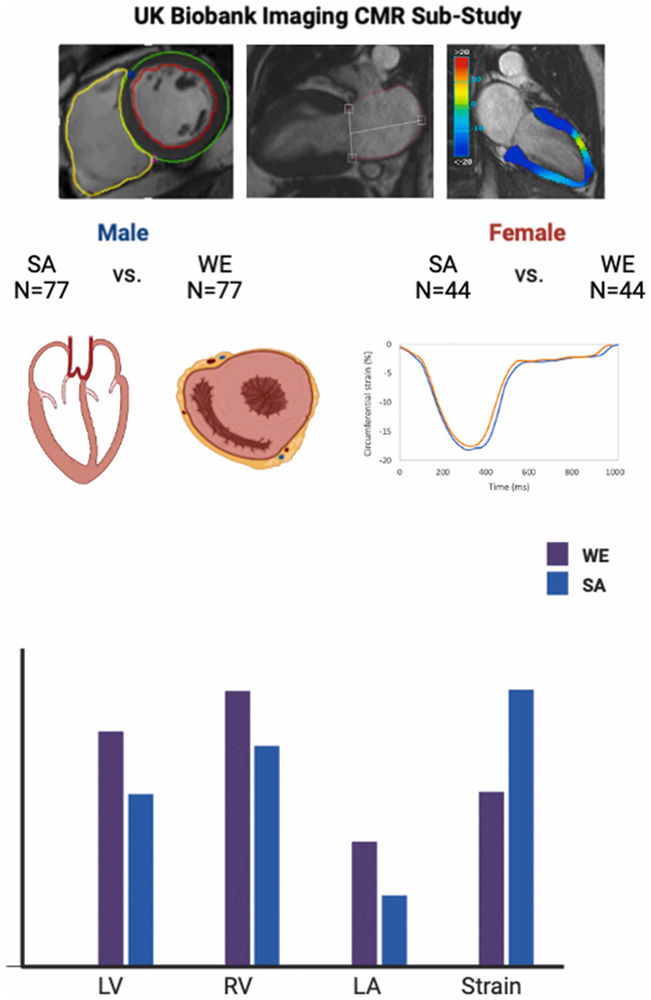
Central illustration. Cardiac MRI-derived ethnic differences in chamber volumes, mass and function between matched South Asian and White Europeans from the UK Biobank Imaging sub-study. In particular, South Asian participants had lower LV mass:volume than WE participants, female but not male South Asian participants had smaller LA volumes than corresponding White European particiapnts, and despite similar LV EF, systolic strain and diastolic strain rates were lower in White European participants.

## Introduction

South Asian people account for almost one quarter of the world population and experience substantially elevated burden of cardiovascular disease. Both atherosclerotic cardiovascular disease and heart failure manifest earlier and with excess morbidity and mortality in South Asian compared with White populations [1], [2]. This is not totally explained by the higher prevalence of risk factors amongst South Asian people, and inherent ethnic differences in cardiac structure and function could potentiate cardiovascular risk. In healthy adults free of prevalent cardiovascular disease and risk factors, ethnic differences in cardiac chamber geometry and function are recognised, which are not entirely attributable to differences in body size [3]. For example, in South Asian compared to Caucasian people, smaller absolute and indexed ventricular volumes, lower left ventricular (LV) mass but higher relative wall thickness and more concentric remodelling, smaller left atrial (LA) dimensions, and higher LV ejection fraction (EF), have been reported [3], [4], [5], [6]. These may account for some of the observed variations in cardiovascular event rates between South Asian and White Europeans groups. However, existing studies in South Asian people have only utilised echocardiography (mainly two-dimensional echocardiography) and focused primarily on LV chamber volumes and EF. None have reported differences in right ventricular (RV) dimensions between White European and South Asian people and no studies have compared ethnic differences in myocardial strain parameters.

Cardiac magnetic resonance imaging (CMR) is the gold-standard imaging modality for assessment of cardiac volumes, mass and EF, and with recent software developments myocardial strain quantitation can be performed from routinely acquired cine images with good agreement with speckle tracking echocardiography [7]. No studies have utilised CMR to compare cardiac chamber geometry and particularly strain between White European and South Asian people.

The UK Biobank imaging study is amongst the world’s largest population-based cohorts with prospectively collected CMR images in middle-aged and older adults, with extensive health questionnaire data and physical measurements [8]. We aimed to compare myocardial mass, volumes and function in healthy adults of White European and South Asian ethnicity matched for age and sex from the UK Biobank. We hypothesised that South Asian people would have smaller cardiac chamber volumes, more LV concentricity, and lower systolic strain, than White European people.

## Methods

### Study population

**Fig. 1:**
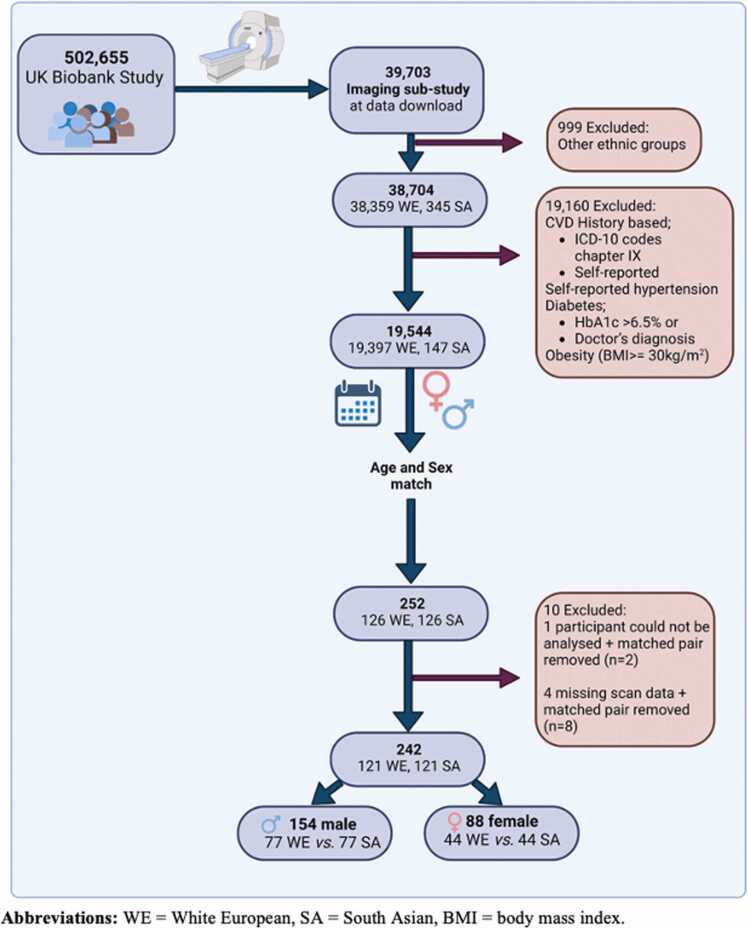
Study profile detailing included participants.

The study flow diagram is provided in Fig. 1. At the point of database interrogation (February 2020), data from 39,703 participants from the UK Biobank CMR sub-study were available for download. Racial and ethnic categories are coded in UK Biobank under the data field 21000 termed “ethnicity” and collected via self-reported touchscreen response at the UK Biobank assessment centre. For the purposes of this study White European people included those who self-reported ethnicity coded as ‘British’, ‘Irish’ or ‘any other white background’ (1001, 1002 and 1003) and South Asian people included those who self-reported ethnicity coded as ‘Indian’, ‘Pakistani’ and ‘Bangladeshi’ (3001, 3002, 3003). To maintain respect, fairness, equality and consistency with participants’ self-reported categories in UK Biobank, the term “ethnicity” is used herein rather than “race and ethnicity” [9]. For identification healthy adults free of cardiovascular or other comorbidities known to impact cardiac chamber geometry and/or function, the following exclusion criteria were applied: cardiovascular disease defined by ICD 10 codes (Chapter IX; Diseases of the circulatory system) from hospital inpatient records up to the time of imaging, in addition to self-reported hypertension, diabetes classified as a HbA1c ≥ 48 mmol/mol (6.5%) or a doctor’s diagnosis, and presence of obesity (defined as BMI ≥ 30 kg/m^2^ irrespective of ethnicity) if recorded prior to *or* at the time of the imaging sub-study. The resultant cohort comprised 19,544 subjects (127 South Asian and 19,397 White European people). Given our primary comparison was between White European and South Asian participants, matching was performed (1:1) based on age and sex using the ‘ccmatch’ program in Stata.

### CMR imaging

The full standardised CMR protocol has been previously published [10]. Briefly, all scans were performed using a wide bore 1.5 T scanner (MAGNETOM Aera, Syngo Platform VD13A, Siemens Healthcare, Erlangen, Germany) at one of the approved UK Biobank participating sites. All images were performed with breath holding. For the purposes of this study, we analysed balanced steady state free precession cine images of long axis (four-, three- and two-chamber) and all short axis slices with complete coverage of the LV and RV. Typical cine parameters were: TR/TE = 2.6/1.1 ms, flip angle 80^0^, Grappa factor 2, voxel size 1.8 × 1.8 × 8 mm (6 mm for long axis), temporal resolution 32 ms with reconstruction to 50 phases per cardiac cycle [10].

### Image analysis

Image analysis was conducted offline and blinded to all participant details by a single experienced observer using Circle CVI (Circle Cardiovascular Imaging, cvi42 v5.13.5 Calgary, Alberta, Canada). All images were assessed for image quality before analysis using the following grading system: 0 =poor (not analysable), 1 =fair (artefacts present but still analysable), 2 = good (artefacts present but not in the region of interest), and 3 =excellent (no artefact). LV function, volumes, wall thickness and mass were calculated using automated rounded epicardial and endocardial borders contoured on the short-axis cine stack at both end diastole and end systole (excluding papillary muscles), with manual checking and correction. The single highest value was reported for end-diastolic wall thickness. LV mass to end-diastolic volume ratio (LV mass:volume) was calculated as a marker of concentric remodelling. Similarly, RV endocardial contours were detected automatically at end diastole and end systole on short-axis cines, again with manual checking and correction as required. To derive maximum and minimum LA volumes, the biplane method was employed using the 4- and 2-chamber long axis cines with manual contouring, excluding the atrial appendage and pulmonary veins. LA EF was derived as (LAVmax-LAVmin) / LAVmax* 100 [27]. Single plane RA minimum and maximum volumes were derived from automated contouring, with manual checking and correction when required, using the 4-chamber long axis view. RA EF was derived as per the formula above but for right atrial values. LV systolic strain and diastolic strain rates were calculated by CMR feature tracking using the cvi42 Tissue Tracking module, to derive longitudinal and circumferential peak early diastolic strain rates (PEDSR), global longitudinal strain (GLS) and global circumferential strain (GCS), as previously described [11], [12]. Manual examination of strain rate curves was undertaken to identify PEDSR, defined as the first peak of the strain rate curve. Radial strain was not quantified due to previous reports of poor reproducibility [25].

### Statistical analysis

Data are reported from point of CMR scanning. Indexing for body surface area (BSA) for cardiac chamber geometry was derived from the Mosteller formula and indexing by height was calculated by dividing relevant imaging parameters by height in meters. Normality was assessed using histograms and the Shapiro-Wilks test. Mean and standard deviation or median and interquartile range were reported, as appropriate. Data is reported by sex and ethnicity with statistical comparisons made between ethnic groups for the given sex via independent T-test or Mann-Whitney U, as appropriate. Linear regression was conducted to adjust for the potential confounders of a history of smoking (current or ex-smoker vs. never smoked) and social deprivation (Townsend index score), with corresponding adjusted *p*-values for ethnicity reported (White European people being the reference group). Those parameters not conventionally indexed to BSA or height were additionally adjusted for height e.g.; stroke volume. LV strain and strain rate represent a relative change in the length of the myocardium and rate of change, respectively. For non-parametric data where residuals were normally distributed raw data were used and where not variables were natural log transformed and then modelled. A *P*-value < 0.05 was considered statistically significant. All values for strain are reported as positive values for ease of interpretation, whereby higher values represent a greater extent of myocardial deformation [13]. Data handling and statistical analyses were performed using StataCorp 2019 (Stata Statistical Software Release 16. StataCorp LLC, College Station, Texas ). Violin plots were generated using GraphPad Prism version 9.0.0 for Windows, GraphPad Software, San Diego, California www.graphpad.com).

## Results

### Study population and participant characteristics

A summary of included participants is displayed in Fig. 1. Following 1:1 matching on ethnicity with sex and age, a total of 254 subjects (127 pairs) were available for inclusion. All subjects whose CMR imaging scored 1–3 on image quality appraisal were included in the analysis. One participant did not have analysable CMR images and five had missing scans. Therefore, each of these participants and their corresponding matched subject were excluded. The final study population comprised 154 males (N = 77 White European and N = 77 South Asian participants) and 88 females (N = 44 White European and N = 44 South Asian participants) (Fig. 1).

Participant characteristics are presented in Table 1. Mean age of the entire cohort was 58 ± 8 years. White European male and female subjects were taller with higher body weight, and accordingly higher BMI and BSA, compared to South Asian male and female subjects. Mean heart rate and blood pressure (BP) were within healthy normal reference ranges [28], although mean systolic BP was higher in White European male participants compared to South Asian male participants. Smoking history was more prevalent in White European male and female participants. There was no significant difference in Townsend score between either ethnicity by sex.

**Table 1:** Participant characteristics.

		Males		Females	
	All	White European	South Asian	White European	South Asian
**n**	**242**	**77**	**77**	**44**	**44**
**Age (years)**	57.9 ± 7.8	57.7 ± 8.0	57.5 ± 8.0	58.7 ± 7.8	58.4 ± 7.6
**Smoking History (n(%))**					
**Never**	180 (75)	46 (60)	67 (87)	27 (61)	40 (91)
**Ex-smoker**	45 (19)	21 (28)	6 (8)	15 (34)	3 (7)
**Current**	16 (6)	9 (12)	4 (5)	2 (5)	1 (2)
**Townsend score**	-1.81 (−3.65, 0.51)	-2.16 (−3.97, 0.29)	-1.33 (−3.078, 0.86)	-2.05 (−3.59, 0.58)	-2.08 (−3.31, 0.28)
**Height (m)**	1.70 ± 0.91	1.78 ± 0.06	1.72 ± 0.01	1.64 ± 0.08	1.59 ± 0.07
**Weight (kg)**	71.2 ± 12.1	80.4 ± 10.3	71.9 ± 9.9	66.5 ± 9.1	58.9 ± 6.9
**BMI (kg/m**^**2**^**)**	24.6 ± 2.7	25.4 ± 2.7	24.3 ± 2.6	24.6 ± 2.9	23.4 ± 2.4
**BSA (m**^**2**^**)**	1.82 ± 0.20	1.99 ± 0.14	1.84 ± 0.14	1.72 ± 0.15	1.60 ± 0.11
**Systolic BP (mmHg)**	125 ± 14	129 ± 13	125 ± 11	121 ± 17	119 ± 15
**Diastolic BP (mmHg)**	78 ± 9	80 ± 8	79 ± 89	74 ± 7	77 ± 10
**Heart rate (bpm)**	67 ± 10	64 ± 9	67 ± 10	67 ± 8	69 ± 10

Data are presented as mean ± SD or median (IQR) as appropriate. Abbreviations: BMI= body mass index, BP =blood pressure, BSA=body surface area.

### Left ventricle

#### Males

**Fig. 2:**
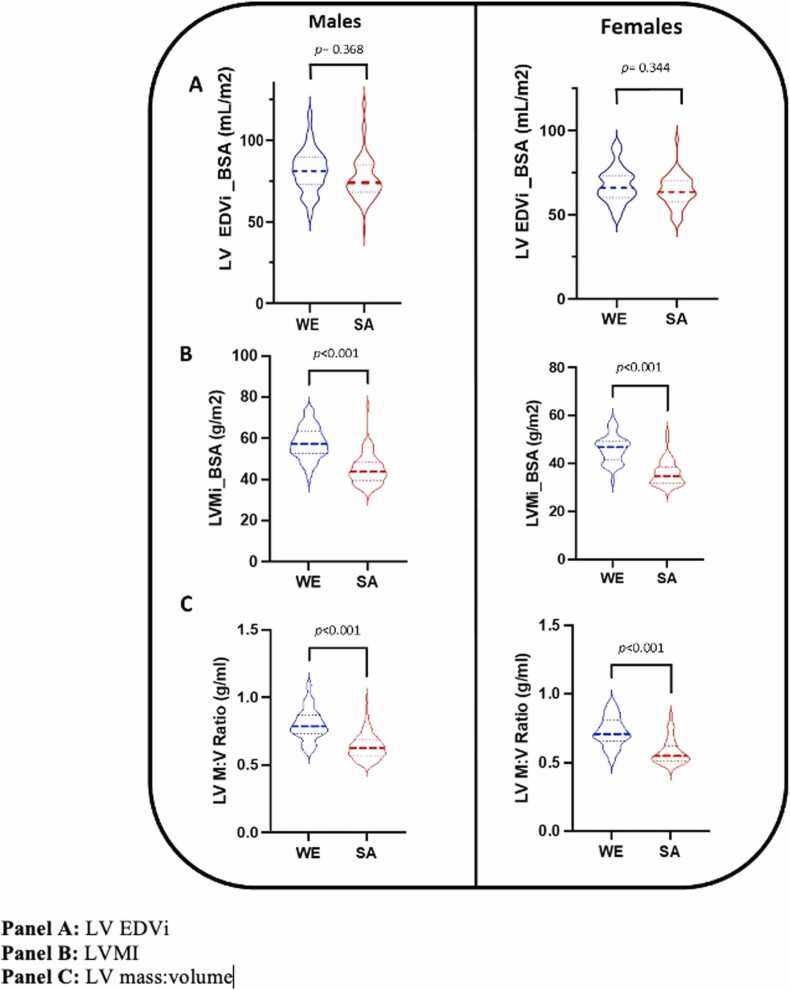
Left ventricular volumes and mass, stratified by sex and ethnicity.

Left ventricular volumes, mass and EF data stratified by sex and ethnicity are presented in Table 2. Compared to White European male subjects, South Asian male subjects had smaller absolute LV end-diastolic (EDV) and end-systolic (ESV) volumes. After indexing to height, South Asian male participants had smaller LV EDV but not ESV. When indexed for BSA, no difference in LV volumes was observed between South Asian and White European males (Fig. 2A). Absolute and indexed LV mass (Fig. 2B) and LV mass:volume (Fig. 2C) were lower in South Asian males than corresponding White European males. No difference in LV EF or cardiac output was observed between South Asian and White European males. End diastolic wall thickness was significantly lower in South Asian compared to White European males, both for absolute values and when indexed to height. All significant differences were independent of smoking status and Townsend score.

**Table 2:** Left ventricular volumes, mass and ejection fraction.

	Males		*p* value	Adjusted *p* value	Females (mean± SD)		*p* value	Adjusted *p* value
	White European	South Asian			White European	South Asian		
Mass(g)*^$^	113.3 (100.2, 130.3)	80.1 (71.5, 93.8)	**< 0.001**	**< 0.001**	78.1 (72.7, 89.7)	56.7 (49.5, 64.0)	**< 0.001**	**< 0.001**
**Mass indexed to BSA (g/m**^**2**^)*	57.2 (52.6, 63.2)	43.7 (39.7, 48.2)	**< 0.001**	**< 0.001**	46.8 (41.9, 49,0)	34.7 (31.9, 38.5)	**< 0.001**	**< 0.001**
Mass indexed to height (g/m)*	63.2 (58.3, 71.4)	46.6 (42.4, 53.6)	**< 0.001**	**< 0.001**	48.4 (44.7, 52.6)	34.9 (32.2, 38.9)	**< 0.001**	**< 0.001**
**EDV (ml)**	145.1 ± 24.8	131.7 ± 25.8	**0.001**	**0.001**	111.7 ± 20.1	101.6 ± 16.0	**0.011**	**0.010**
**EDVi BSA (ml/m**^**2**^**)**	72.7 ± 11.1	70.1 ± 11.9	0.368	0.181	64.6 ± 9.9	62.7 ± 7.9	0.344	0.384
**EDVi height (ml/m)**	81.6 ± 13.0	76.6 ± 13.6	**0.022**	**0.012**	67.7 ± 10.5	63.9 ± 9.3	0.075	0.082
**ED wall thickness (mm)**^**$**^	8.9 (8.4, 10.2)	8.1(7.6, 9.1)	**< 0.001**	**0.001**	7.2 (6.5, 8.0)	6.7 (6.1, 7.8)	0.066	0.138
**ED wall thickness i height (mm/m)**^**$**^	5.1 (4.6, 5.7)	4.7 (4.4, 5.3)	**0.005**	**0.049**	4.4 (4.0, 5.0)	4.2 (3.9, 5.0)	0.413	0.601
ESV (ml)*	57.3 ± 14.2	52.3 ± 14.6	**0.035**	**0.025**	37.0 (32.1, 43.5)	36.9 (29.6, 43.0)	0.427	0.261
**ESVi BSA (ml/m**^**2**^)*	28.7 ± 6.7	28.2 ± 7.1	0.661	0.505	22.4 (18.4, 25.3)	23.0 (19.7, 25.4)	0.483	0.759
ESVi height (ml/m)*	32.2 ± 7.7	30.4 ± 8.0	0.164	0.135	22.7 (19.5, 26.4)	22.5 (19.6, 26.5)	0.839	0.708
**SV (ml)**	87.8 ± 15.1	79.4 ± 14.7	**< 0.001**	0.346	72.7 ± 14.6	64.6 ± 10.5	**0.004**	0.208
**EF (%)**	61.2 ± 4.9	60.6 ± 5.7	0.508	0.487	65.1 ± 5.4	63.8 ± 5.0	0. 225	0.291
Mass:volume (g/ml)*	0.79 (0.73, 0.87)	0.63 (0.57, 0.69)	**< 0.001**	**< 0.001**	0.71 (0.66, 0.81)	0.54 (0.51, 0.63)	**< 0.001**	**< 0.001**
CO (L/min)*	5.00 (4.40, 5.60)	4.30 (3.80, 4.70)	**< 0.001**	**< 0.001**	4.11 ± 0.92	3.60 ± 0.64	**0.004**	0.351
CO indexed BSA (L/min/m2)*	2.51 (2.24, 2.94)	2.53 (2.24, 2.82)	0.567	0.207	2.52 ± 0.41	2.58 ± 0.44	0.500	0.407

Adjusted p-values represent comparison between South Asian and White European participant groups after adjustment for smoking status and Townsend deprivation index. Abbreviations: LV = left ventricle, EDV = end diastolic volume, i = indexed, BSA = body surface area, ESV = end systolic volume, SV= stroke volume, EF= ejection fraction, CO = cardiac output. *data non-normally distributed (median and interquartile range reported). $ = Log transformed data prior to multivariate linear regression

#### Females

Left ventricular volumes, mass and EF in females, stratified by ethnicity, are presented in Table 2. South Asian female subjects demonstrated lower absolute and indexed LV mass (Fig. 2B), and lower absolute EDV, independent of smoking status and Townsend score. However, no difference was observed in EDV when indexed (BSA and height), (Fig. 2A) as well as for absolute or indexed (BSA and height) ESV nor end diastolic wall thickness. Ejection fraction and cardiac index were similar. LV mass:volume (Fig. 2C) was lower in South Asian than White European female participants, remaining so after adjustment for smoking status and Townsend score.

### Right ventricle

#### Males

**Fig. 3:**
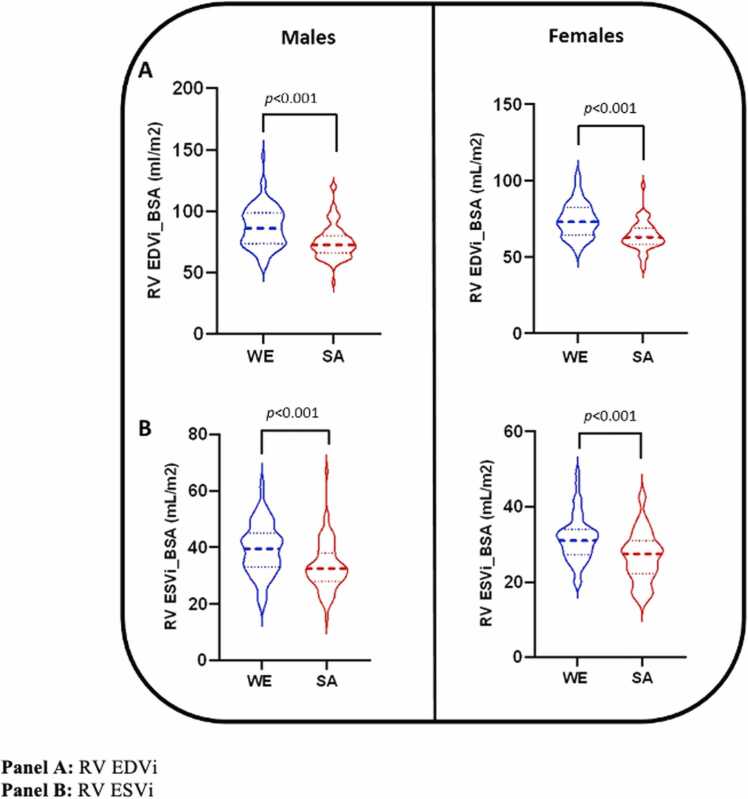
Right ventricular volumes, stratified by sex and ethnicity.

Male RV volumes and EF stratified by ethnicity are presented in Table 3 and Fig. 3A and B. Both absolute and indexed values for RV EDV and ESV were consistently lower overall in South Asian than White European male subjects independent of smoking status and Townsend score. Although there was no difference in EF between ethnic groups, South Asian male participants had significantly lower stroke volume than White European male participants, which remained after adjusting for smoking status, Townsend score and height.

**Table 3:** Right ventricular volumes and ejection fraction.

	Males		*p* value	Adjusted *p* value	Females		*p* value	Adjusted *p* value
	White European	South Asian			White European	South Asian		
**EDV (ml)**	174.0 ± 35.2	139.1 ± 30.3	**< 0.001**	**< 0.001**	128.0 ± 21.3	103.5 ± 20.4	**< 0.001**	**< 0.001**
**EDVi BSA (ml/m**^**2**^**)**	87.3 ± 16.6	75.0 ± 13.8	**< 0.001**	**< 0.001**	74.1 ± 11.3	63.8 ± 9.5	**< 0.001**	**< 0.001**
**EDVi height (ml/m)**	97.8 ± 18.4	80.9 ± 16.2	**< 0.001**	**< 0.001**	77.7 ± 11.8	65.2 ± 13.2	**< 0.001**	**< 0.001**
ESV (ml)*	78.4 ± 18.5	61.9 ± 17.2	**< 0.001**	**< 0.001**	54.0 (46.5, 63.1)	44.5 (35.0, 49.8)	**< 0.001**	**< 0.001**
**ESVi BSA (ml/m**^**2**^)*	39.3 ± 9.1	33.4 ± 8.6	**< 0.001**	**< 0.001**	31.4 (27.6, 33.8)	27.4 (22.5, 31.1)	**< 0.001**	**< 0.001**
ESVi height (ml/m)*	44.1 ± 10.1	36.0 ± 9.6	**< 0.001**	**< 0.001**	33.1 (28.8, 37.4)	27.6 (22.5, 31.5)	**< 0.001**	**< 0.001**
SV (ml)*	91.4 (80.1, 111.9)	75.3 (67.0, 84.2)	**< 0.001**	**0.002**	74.2 (61.5, 82.6)	59.2 (50.5, 68.5)	**< 0.001**	**< 0.001**
**EF (%)**	55.0 ± 6.2	55.9 ± 5.4	0.357	0.538	57.3 ± 5.4	58.3 ± 7.5	0.496	0.678

Adjusted p-values represent comparison between South Asian and White European participant groups after adjustment for smoking status and Townsend deprivation index. Abbreviations: RV = right ventricle, EDV = end diastolic volume, i = indexed, BSA = body surface area, ESV = end systolic volume, SV = stroke volume, EF = ejection fraction. *data non-normally distributed (median and interquartile range reported).

#### Females

In females, similar ethnic differences in RV volumes and EF were observed as those seen in males (Table 3). South Asian female subjects had lower overall absolute and indexed RV volumes than White European female subjects, even after adjustment for smoking status and Townsend score. There were no differences in EF.

#### Left atrium

No difference in LA volumes or EF were observed between ethnic groups in males (Table 4). In contrast, South Asian female participants had lower absolute and indexed LA volumes independent of smoking status, Townsend score and height and lower LA EF compared to White European female participants.

**Table 4:** Left and right atrial volumes and ejection fraction.

	Males		*p* value	Adjusted *p* value	Females		*p* value	Adjusted *p* value
	White European	South Asian			White European	South Asian		
**LA maximum V (ml)**	60.7 ± 16.6	56.7 ± 16.4	0.152	0.091	56.3 ± 16.3	45.9 ± 10.5	**< 0.001**	**0.001**
**LA maximum Vi BSA (ml/m**^**2**^**)**	30.4 ± 7.9	30.6 ± 7.8	0.887	0.823	32.7 ± 9.3	28.8 ± 6.2	**0.025**	**0.029**
**LA maximum Vi height (ml/m)**	34.1 ± 9.1	32.9 ± 9.1	0.438	0.304	34.1 ± 9.7	28.9 ± 6.4	**0.005**	**0.005**
**LA EF (%)**	60.4 ± 16.6	56.3 ± 16.3	0.153	0.092	56.0 ± 16.3	46.0 ± 10.5	**< 0.001**	**0.001**
**RA maximum V (ml)**	75.8 (65.6, 95.7)	63.6 (52.7, 74.9)	< 0.001	< 0.001	64.5 (55.8, 71.8)	49.4 (42.6, 62.1)	**< 0.001**	**< 0.001**
**RA maximum Vi BSA (ml/m**^**2**^**)**	39.3 (33.2, 47.9)	35.7 (30.9, 40.4)	0.012	0.004	37.0 (32.3, 40.8)	32.1 (27.1, 38.7)	**< 0.001**	**0.004**
**RA maximum Vi height (ml/m)**	43.7 (36.5, 53.0)	37.9 (31.1, 44.0)	0.002	< 0.001	38.3 (34.1, 43.7)	31.9 (27.3, 38.4)	**< 0.001**	**0.004**
**RA EF (%)**	49.3 ± 6.6	51.3 ± 8.5	0.106	0.024	55.6 ± 7.7	55.2 ± 6.6	**0.789**	**0.749**

Adjusted p-values represent comparison between South Asian and White European participant groups after adjustment for smoking status and Townsend deprivation index. Abbreviations: LA = left atrium, V = volume, i = indexed, BSA = body surface area, RA= right atrium. $ = Log transformed data prior to multivariate linear regression

#### Right atrium

Both absolute and indexed RA volumes were lower in South Asian compared White European males and female participants. In males, RA EF was significantly higher in South Asian compared to White European participants, but there was no difference in RA EF between South Asian and White European female participants (Table 4).

### Left ventricular strain and strain rates

#### Males

**Fig. 4:**
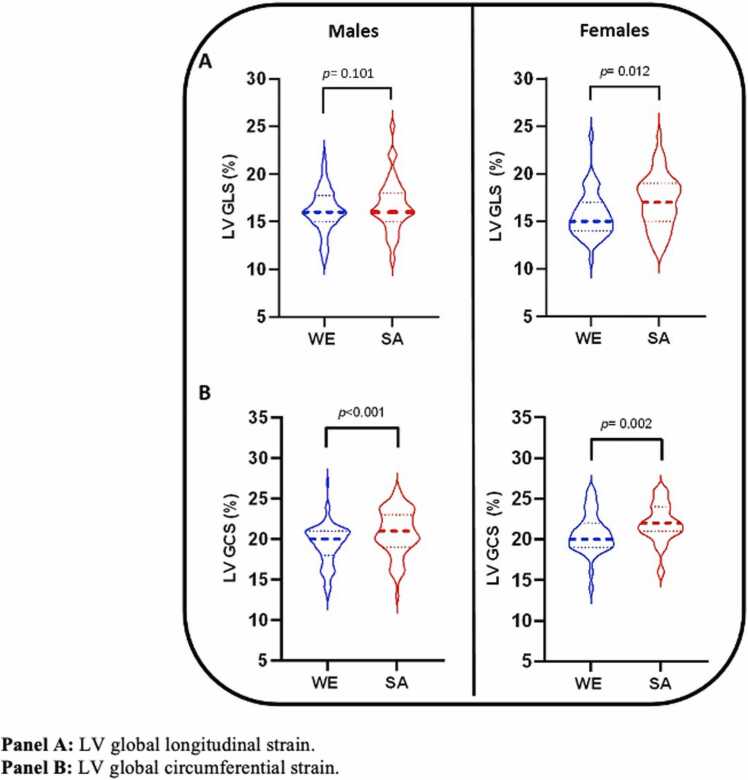
Left ventricular systolic strain, stratified by sex and ethnicity.

South Asian male participants had higher GCS but not GLS compared to WE male participants (Table 5, Fig. 4A and B), which remained significant after adjustment for smoking status and Townsend score. Similarly, both circumferential and longitudinal PEDSR were higher in South Asian than White European male subjects in both the unadjusted and adjusted models (Table 5).

**Table 5:** Left ventricular strain and strain rates.

	Males		*p* value	Adjusted *p* value	Females		*p* value	Adjusted *p* value
	White European	South Asian			White European	South Asian		
**LV GLS**	16.1 ± 2.2	16.8 ± 2.7	0.101	**0.246**	15.7 ± 2.3	17.1 ± 2.7	**0.012**	0.058
**LV GCS**	19.3 ± 2.5	20.8 ± 2.8	**< 0.001**	**0.001**	20.4 (19.2, 21.9)*	21.9 (20.8, 23.6 *)	**0.003**	0.072
**LongPEDSR (s**^**-1**^**)**	0.73 ± 0.19	0.84 ± 019	**< 0.001**	**0.003**	0.86 ± 0.20	0.94 ± 0.32	0.099	0.195
**CircPEDSR (s**^**-1**^**)**	0.94 ± 0.21	1.05 ± 0.23	**0.002**	**0.029**	1.14 ± 0.25	1.19 ± 0.32	0.390	0.579

Adjusted p-values represent comparison between South Asian and White European participant groups after adjustment for smoking status and Townsend deprivation index. **Abbreviations:** GLS = global longitudinal strain, GCS = global circumferential strain, LongPEDSR = longitudinal peak early diastolic strain rate, CircPEDSR = circumferential peak early diastolic strain rate.* Median and interquartile range reported for non-parametric data

#### Females

Both GLS and GCS were higher in South Asian female compared to WE female participants (Table 5, Fig. 4A and B); however, the statistical significance of these differences diminished after adjustment for smoking status and Townsend score. No differences in longitudinal or circumferential PEDSR were observed between White European and South Asian female participants.

## Discussion

To our knowledge this is the first study utilising CMR to compare ethnic differences in cardiac structure and function, including strain, between middle-aged healthy White European and South Asian people. We demonstrate ethnic differences in cardiac chamber volumes, mass, and systolic strain and diastolic strain rates. Male and female South Asian people had smaller LV and RV volumes, lower LV mass and contrary to the prevailing literature, lower LV mass:volume, compared to White European people. LV end-diastolic wall thickness was lower in male South Asian than White European people, but no ethnic difference was observed between females. Additionally, male and female South Asian people had smaller RA volumes than White European people, but only female South Asian subjects had smaller LA volumes than corresponding White European subjects. Despite South Asian and White European people having similar LV and RV EF, LV GLS, GCS and PEDSR were lower in White European people, with variations according to sex. These findings could have implications for normative reference ranges, clinical and research image interpretation.

Few previous studies have specifically compared cardiac chamber volumes and function between healthy South Asian people and other ethnic groups, and only using two-dimensional echocardiography. Amongst the first of these was the LOLIPOP population-based study, which examined 259 South Asian and 199 White European adults, aged 35–74 years, free from traditional cardiovascular risk factors and excluding those with subclinical coronary atherosclerosis (computed tomography coronary calcium score >10 Agatston units) [4]. South Asian people had significantly reduced LV and LA volumes and lower LV mass than White European people, even when corrected for body size. However, in the LOLIPOP cohort these changes were associated with increased relative wall thickness, a measure of concentric remodelling, among South Asian people [4], [5]. Confirmation of these findings was made in the subsequent larger Echocardiographic Normal Ranges Meta-Analysis of the Left Heart (EchoNoRMAL) collaboration, which was a combined population-based dataset comprising echocardiographic measurements from over 22,000 adults (comprising 14,042 White European and 1751 South Asian people) free of cardiovascular disease, renal disease, hypertension or diabetes. EchoNoRMAL similarly described South Asian people as having smaller absolute and indexed LV EDV and ESV, lower LV mass but higher relative wall thickness, smaller LA dimensions, and higher LV EF, compared to White European people [3]. When three-dimensional echocardiography was used to compare White European (N = 427) with South Asian (N = 325) adults aged 55–85 years in the Southall and Brent Revisited (SABRE) study, however, no difference in relative wall thickness was observed and LV remodelling index was actually lower in South Asian people, although this cohort included ∼40% with diabetes and ∼1/4 with coronary disease [6].

In our healthy cohort from UK Biobank using CMR, we also found smaller cardiac chamber volumes and lower LV mass in South Asian participants, but with no differences in EF. A striking difference to EchoNoRMAL and LOLIPOP, however, was our finding that South Asian participants also had ∼20% lower LV mass:volume and 5–10% lower end-diastolic wall thickness (but only in male subjects) compared to White European participants. Because both LV mass and EDV were lower in South Asian people, it is reasonable to expect that LV concentricity would be similar to White European people. This was not, however, the case in our study and it appears that the lower LV mass:volume in South Asian people was driven by a disproportionately lower LV mass and wall thickness than EDV. This discrepancy between studies may be due to past reliance on the linear two-dimensional echocardiographic measurement of relative wall thickness, which only utilises diastolic basal septal and posterior wall thickness together with LV diameter, is reliant on the acoustic window angle and may not reflective of true LV geometry [14]. Compared to transthoracic echocardiography, evaluation by CMR using contiguous short axis slices covering the extent of the LV and is free of geometric assumptions. We are confident in our finding that LV mass:volume is lower in South Asian people, supported by our finding of lower wall thickness in SA participants. This may challenge the notion that healthy South Asian people have more concentric LV hypertrophy than White European people, which has previously been attributed as a harbinger of excess cardiovascular risk in South Asian populations [15]. Perhaps the excess cardiovascular risk in South Asian people related to concentric LV remodelling occurs as a result of a heightened impact of hypertension and other cardiometabolic traits on LV hypertrophy compared to White European people, which has been described in comparisons of South Asian men and women [16]. However, we acknowledge that several systematic differences between ethnic groups, which may distinguish South Asian from White European people (such as dietary intake, physical activity levels, occupational and environmental exposures), were not adjusted for in our comparisons and could have impacted cardiac structure and function.

In addition to the observed ethnic-related LV remodelling patterns, ours is the first study to describe differences in multidimensional LV systolic strain and diastolic strain rates between South Asian and White European people. Using CMR feature tracking, we found in both men and women that GCS was higher in South Asian participants, and additionally in women that GLS was higher in South Asian than in White European subjects (although not adjusted values), despite similar LV EF across groups. These observed differences in systolic strain may be related to variations in LV wall thickness and concentric remodelling between ethnicities, as LV mass:volume has previously been shown to have an inverse relationship with peak systolic circumferential strain in a subset (N = 441) of participants from the Multi-Ethnic Study of Atherosclerosis (MESA) who underwent tagged CMR [17]. In men but not women, PEDSR was also higher in South Asian than in White European people, again in contrast to the LOLIPOP study where mitral annular systolic and diastolic velocities were lower in South Asian people [4].

Strain measurement enables multiplanar assessment of global and regional myocardial contraction and relaxation, obviating many of the limitations of EF [18], [19]. Multiple studies have demonstrated incremental diagnostic and prognostic utility of myocardial strain evaluation across disease states [20], [21], [22]. Indeed, myocardial strain evaluation (particularly GLS) is advocated for diagnosis in numerous contemporary European Society of Cardiology guidelines, including heart failure [23], coronary artery disease [24], valvular heart disease [25], and cardio-oncology [26]. Existing cut-offs (typically GLS less than 16% being considered abnormal) were originally derived from speckle tracking echocardiography studies in healthy volunteers and did not take into account ethnic differences that may impact strain measurements in health and disease [27]. In a meta-analysis of 24 studies comprising 2597 apparently healthy subjects, wide variations in strain derived from speckle tracking echocardiography were observed (e.g. reported normal values for GLS were between 15.9 to 22.1%) [28]. More recently in the largest meta-analysis of CMR feature tracking studies, comprising 3359 healthy adults, normal reference values for LV GLS (18.4%; 95% CI 17.6 to 19.2%) and GCS (21.4%; 95% CI 20.6 to 22.3%) have been proposed [29]. However, these are considerably higher than the strain values observed in our UK Biobank cohort, although this meta-analysis incorporated studies using different CMR scanner platforms, fields strengths, and feature tracking strain software vendors, and again found wide variations in mean strain values reported between included studies. Importantly in both these meta-analyses ethnicity remained an unmeasured confounder. Our findings suggest the existence of ethnicity-related variations in myocardial strain assessment, but we lack sufficient power to adjust for multiple potential confounders and define normographic reference ranges, and the clinical impact of these differences is not known. Further work is needed to establish robust ethnic-specific strain cut-offs in health and disease states. Although no reference gold-standard technique for strain assessment exists, in contemporary clinical practice myocardial strain is measured primarily using echocardiography and not CMR feature tracking. Validation of our ethnic-related differences in strain using echocardiography is therefore warranted.

In a similar pattern to LV volumes, we found that South Asian men and women had smaller absolute and indexed RV volumes, with comparable RV EF. Sex and ethnic differences in RV morphology assessed by CMR were best described in the Multi-Ethnic Study of Atherosclerosis (MESA) study, which defined RV mass, volumes and EF in healthy adults but did not include South Asian participants [30]. To our knowledge, ours is the first study to demonstrate lower RV volumes in South Asian than White European people, which were more pronounced than differences in LV volumes. For example, LV EDV was ∼10% lower in South Asian than White European participants, whereas RV EDV was almost 20% lower in male South Asian than White European subjects. The same pattern of ethnic difference in RV volumes was observed amongst women in our cohort. The clinical impact of these differences in RV volumes is unclear, but again highlights a need for ethnic-specific CMR normative reference ranges, which do not currently exist.

Indexed LA volumes were smaller in South Asian women, but not men, compared to White European women, in this study. By contrast, absolute and indexed RA volumes were smaller in both South Asian men and women compared to White European men and women. These small inconsistencies between ethnic differences in LA and RA volumes likely occurred due to differences in quantitation method (i.e. single plane for RA and biplane for LA volumes) and it is unclear why ethnicity would differentially impact geometry of the RA and LA. Prior to this study, ethnic differences in LA volumes in White, Chinese-American, Black and Hispanic race and ethnic groups were best demonstrated using CMR in MESA [31]. To our knowledge, only one small retrospective study (N = 120, 50% SA, 50% males) specifically evaluated differences in LA volumes between South Asian and White European people, which also noted smaller LA volumes in South Asian subjects albeit in patients referred for clinical CMR [32]. RA volumes were not reported in this study and no association between sex and LA volumes were observed, although the small sample size did not permit robust sex comparisons and the prevalence of risk factors known to impact LA volumes (such as diabetes and hypertension) were additional confounders [32]. Given LA (and more recently RA) enlargement is increasingly being recognised for its association with adverse outcomes, including coronary artery disease, heart failure, stroke, all-cause and cardiovascular death, and particularly atrial fibrillation, ethnic differences in LA volumes may contribute to variations in cardiovascular risk [33], [34], [35]. For example, South Asian people have consistently been found to have lower risk of developing atrial fibrillation even with higher risk factor burden, although interestingly, White European females were found to have lower risk of AF compared to South Asian females in one large (N = 277,218, 26% SA) cohort study [36]. Clearly the relationship between LA structure and incident cardiovascular disease warrants prospective evaluation with adjustment for ethnicity.

## Strengths and limitations

Key strengths of the study are the careful matching for age and sex of South Asian and White European people, exclusion of participants with established cardiovascular disease and risk factors, and assessment of cardiac structure and function (including strain) using gold-standard CMR. Limitations include the modest sample size, which was restricted due to the UK Biobank cohort predominantly comprising White European participants with further attrition of subjects after close matching of ethnic groups, and lack of validation with echocardiography. The interaction between age strata, ethnicity and cardiovascular structure/function was not assessed for the same reason, and the inclusion in UK Biobank only of adults aged 45–74 years is another limitation. Physical activity levels, which are known to impact cardiac geometry, were not compared between ethnic groups because of a significant amount of missing data for self-reported levels and not having access to the UK Biobank accelerometer sub-study data for an objective measure. Additional confounders, such as dietary intake, occupation and other environmental exposures were accounted for due to lack of data availability. A further limitation pertains to not exploring any potential genetic associations for observed differences in cardiac structure and function between the groups a decision that was driven by the small sample size. Lastly, other ethnic groups were not included in this study, although we purposely sought to focus on South Asian people due to the paucity of data in this group.

## Conclusions

In this comparison of closely matched healthy White European and South Asian participants from the UK Biobank Imaging CMR sub-study, we identified ethnic differences in cardiac structure and function. South Asian participants had lower LV mass:volume, end-diastolic wall thickness and smaller atrial volumes than corresponding White European participants. Despite similar LV EF, systolic strain and diastolic strain rates were lower in White European participants. Large-scale studies are needed to clearly define ethnic-specific normative reference ranges, with stratification for age and sex.

## Ethical approval and consent to participate

UK Biobank has approval from the North West Multi-centre Research Ethics Committee as a Research Tissue Bank. All participants provided written informed consent.

## Consent for publication

Not applicable.

## Funding

EMB funded by the National Institute for Health Research (10.13039/100006662NIHR) through a Research Professorship award (McCann, RP-2017-08-ST2-007) and directly supported by the Leicester NIHR Biomedical Research Centre and Clinical Research Facility. JMB is funded by a Research Fellowship Award from the 10.13039/100011056British Society for Heart Failure. AD received funding from the 10.13039/501100000274British Heart Foundation through a Clinical Research Training Fellowship (FS/CRTF/20/24069). The remaining authors have no funding to report.

## Author contributions

KSP performed the CMR image analysis and managed the study database. EMB undertook the statistical analysis and oversaw day-to-day coordination of the study. AA oversaw the LA analysis. RSM and CSR undertook UK Biobank data selection and download. AS, JRA, MPMGB, JMB, SLA, AD and JLY critically reviewed the data and manuscript draft. GPM conceived the idea for the study and designed the protocol. GSG drafted the manuscript. All authors read and approved the final manuscript.

## CRediT authorship contribution statement

**Singh Anvesha:** Supervision. **Arnold Jayanth R.:** Supervision. **Graham-Brown Matthew PM:** Project administration, Visualization, Writing – original draft. **Bilak Joanna M:** Data curation, Project administration, Visualization. **Ayton Sarah L:** Methodology, Software, Visualization. **Dattani Abhishek:** Investigation, Supervision, Writing – original draft, Writing – review & editing. **Gulsin Gaurav S:** Conceptualization, Data curation, Formal analysis, Investigation, Methodology, Project administration, Supervision, Visualization, Writing – original draft, Writing – review & editing. **Yeo Jian L.:** Investigation, Supervision, Writing – original draft, Writing – review & editing. **Parke Kelly S.:** Data curation, Formal analysis, Methodology, Writing – original draft, Writing – review & editing. **McCann Gerry P.:** Conceptualization, Formal analysis, Funding acquisition, Investigation, Methodology, Project administration, Resources, Supervision, Writing – review & editing. **Brady Emer M.:** Conceptualization, Data curation, Formal analysis, Investigation, Methodology, Supervision, Writing – original draft, Writing – review & editing. **Alfuhied Aseel:** Formal analysis, Investigation, Methodology. **Motiwale Rishabh S.:** Data curation, Formal analysis. **Razieh Cameron S.:** Formal analysis.

## Declaration of Competing Interest

The authors declare that they have no competing interests.

## Data Availability

The imaging datasets analysed for the current study are available in the UK Biobank repository. The resulting quantitative imaging data generated during the current study are available from the corresponding author on reasonable request.
